# Clear cell endometrial carcinoma with high microsatellite instability in a complicated pregnancy: a case report

**DOI:** 10.1186/s13256-023-03994-y

**Published:** 2023-07-09

**Authors:** Fabian Weiss, Till Kaltofen, Veronika Kanitz, Lennard Schröder, Bernd Kost, Alexander König, Maria Delius, Sven Mahner, Irene Alba Alejandre

**Affiliations:** 1 Department of Obstetrics and Gynecology, University Hospital, Ludwig-Maximilians-University, Marchioninistrasse 15, 81377 Munich, Germany; 2 Department of Surgery, University Hospital Regensburg, Regensburg, Germany; 3 Institute of Pathology, Faculty of Medicine, Ludwig-Maximilians-University, Munich, Germany

**Keywords:** Endometrial neoplasms, Pregnancy, DNA mismatch repair, Clear cell adenocarcinoma, Amniocentesis, Case report

## Abstract

**Background:**

Endometrial carcinomas are the most common female genital malignancies. They are very rare in pregnancy and worldwide less than 60 cases associated with pregnancy are published. No clear cell carcinoma has been described in a pregnancy with a live birth.

**Case presentation:**

We present the course of a 43-year-old Uyghur female patient with the diagnosis of endometrial carcinoma with a deficiency in the DNA mismatch repair system in the pregnancy. The malignancy with clear cell histology was confirmed by biopsy following the delivery via caesarean section due to preterm birth of a fetus with sonographically suspected tetralogy of Fallot. Earlier whole exome sequencing after amniocentesis had shown a heterozygous mutation in the MSH2 gene, which was unlikely to be related to the fetal cardiac defect. The uterine mass was initially deemed an isthmocervical fibroid by ultrasound and was confirmed as stage II endometrial carcinoma. The patient was consequently treated with surgery, radiotherapy and chemotherapy. Six months after the adjuvant therapy, re-laparotomy was performed due to ileus symptoms and an ileum metastasis was found. The patient is currently undergoing immune checkpoint inhibitor therapy with pembrolizumab.

**Conclusion:**

Rare endometrial carcinoma should be included in the differential diagnosis of uterine masses in pregnant women with risk factors.

## Background

Endometrial carcinoma (EC) is the most common genital carcinoma in women in high-income countries with a cumulative risk of 1% by age 75 [1]. While it usually is a cancer in postmenopausal females, up to 25% occur in premenopausal women [1, 2]. Whereas cervix, ovarian or breast cancer is more common in pregnancy [3], endometrial carcinoma in this situation is rare: Worldwide no more than 55 pregnancy-related cases have been published since 1927 [4–8]. Most of these pregnancy-related ECs are diagnosed through dilatation and curettage and are low grade endometrioid carcinomas.

The prevalent subtype of endometrial cancer is estrogen-dependent endometrioid cancer, which has a better prognosis than non-endometrioid cancer such as serous or clear cell carcinoma. Recently a molecular profiling has been established besides traditional histologic subtypes: This new classification reflects genetic aberrations and clinical behavior; namely POLE, microsatellite instable, copy-number-high and copy-number-low tumors [9, 10]. ECs with microsatellite instability (MSI) due to a deficiency in functional mismatch repair proteins (dMMR) such as MLH1, MSH2, MSH6, PMS2 [11] account for 30% of endometrial carcinomas [12, 13]. These mismatch repair proteins correct errors during DNA replication [14]. Resulting deficiencies in this repair system increase the possibility of accumulating gene mutations, especially in conserved repetitive DNA regions called microsatellites. Here, aggregations of mutations lead to microsatellite instability (MSI) [14, 15]. Therefore a deficiency in mismatch repair proteins (dMMR) leads to hypermutation and accelerates carcinogenesis [14], especially for colon and endometrial cancer [16]. If MMR-mutations are germline-located, they provoke an elevated hereditary risk for these cancers—namely, the autosomal dominant inherited Lynch-Syndrome [1, 16, 17].

The molecular classification usually is applied after diagnosis of cancer and guides treatment approaches [18] but we present a case where prenatal testing could have pointed towards subsequent diagnosis of endometrial carcinoma with microsatellite instability in pregnancy.

## Case presentation

A 43-year-old Uyghur woman presented herself to our emergency obstetric department with menstruation-like vaginal bleeding in her 5^th^ pregnancy with 24 6/7 weeks of gestation. The patient had conceived spontaneously. In the obstetrical history, the patient had had one vaginal birth and three cesarean deliveries in the last 13 years. Two of the deliveries were late preterm and one was a twin pregnancy. All children were healthy. During the last two pregnancies she suffered from gestational diabetes. Obesity with a BMI of 42 kg/m^2^ and status after laparoscopic cholecystectomy were the only relevant comorbidities. The last cervix PAP smear from the first trimester showed a result without any abnormalities.

A detailed second trimester ultrasound had been performed in an external prenatal care diagnostic clinic 3 weeks prior to the first in house presentation. A tetralogy of Fallot was suspected in the fetal echocardiography. To rule out a genetic syndrome, an amniocentesis was performed externally and showed an unremarkable male karyotyping. In the further molecular workup through whole exome sequencing, a heterozygous pathogen mutation in the MSH2 gene (c.560T > C p.(Leu187Pro)) was detected, which was not considered to be related to the cardiac anomaly.

**Fig. 1:**
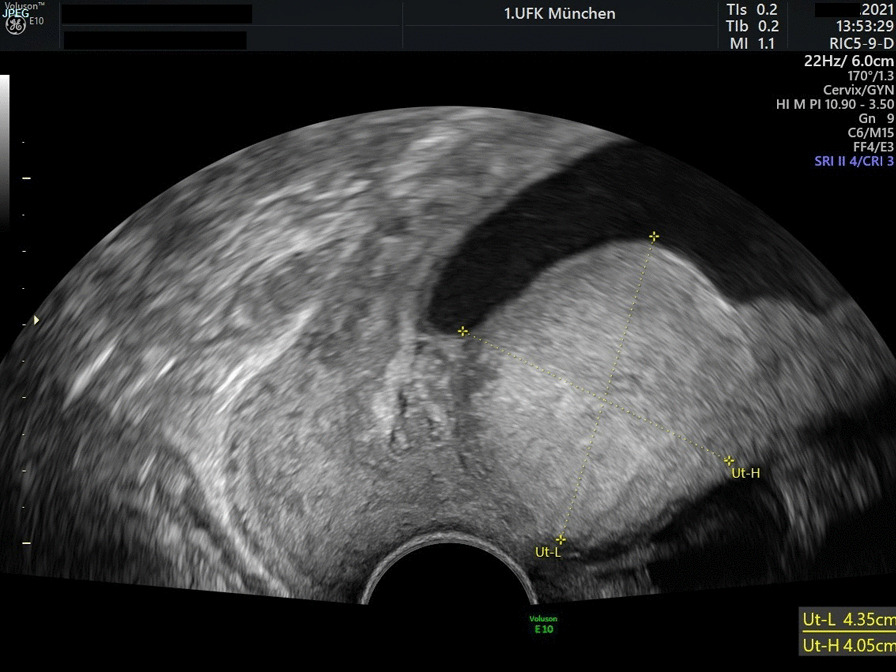
Transvaginal ultrasound with uterine mass. Transvaginal ultrasound of the cervix and uterine isthmus on admission of the patient with 24 6/7 weeks of gestation. The mass was initially assessed as a fibroid but was later confirmed as a clear cell endometrial carcinoma in the pregnancy via obtaining a specimen at caesarean section

When the patient presented herself to our clinic with 24 6/7 weeks of gestation, the cervix was 27 mm long, the fetus showed growth according to gestational age and normal Doppler values. On transvaginal ultrasound, a 4.8 cm mass close to the cervix was interpreted as a fibroid, shown in Fig. 1. Due to the preterm bleeding, we started respiratory distress prophylaxis with 12 mg betamethasone i.m., tocolysis with the oxytocin-receptor antagonist atosiban and antibiotics with ampicillin and sulbactam i.v. Bacterial testing revealed ureaplasma parvum and antibiotic treatment was expanded to azithromycin orally. The bleeding regressed.

One week after admission (25 6/7 weeks of gestation), the patient stated increased contractions. An examination showed a three centimeter dilated cervix with a prolapse of the membranes. Laboratory and clinical testing did not show any sign for acute infection. A McDonald rescue cerclage was performed without any complications after thorough informed consent due to imminent preterm birth with the suspected cardiac defect. Perioperative tocolysis with indomethacin was applied. With 26 4/7 weeks of gestation, we observed a recurrence of the bleeding and a new prolapse of the membranes. We indicated an emergency caesarean section.

Intraoperative evaluation of the suspected fibroid, showed necrotizing—extremely vulnerable—tissue in the lower uterine segment. This was removed as completely as possible and sent to histology.

The preterm male, 980 g (APGAR 5/7/9) was admitted to the neonatal intensive care unit after intubation due to respiratory distress. The tetralogy of Fallot was confirmed by echocardiography. Unfortunately, on day 27 the newborn died from a fulminant sepsis due to necrotizing enterocolitis.

**Fig. 2:**
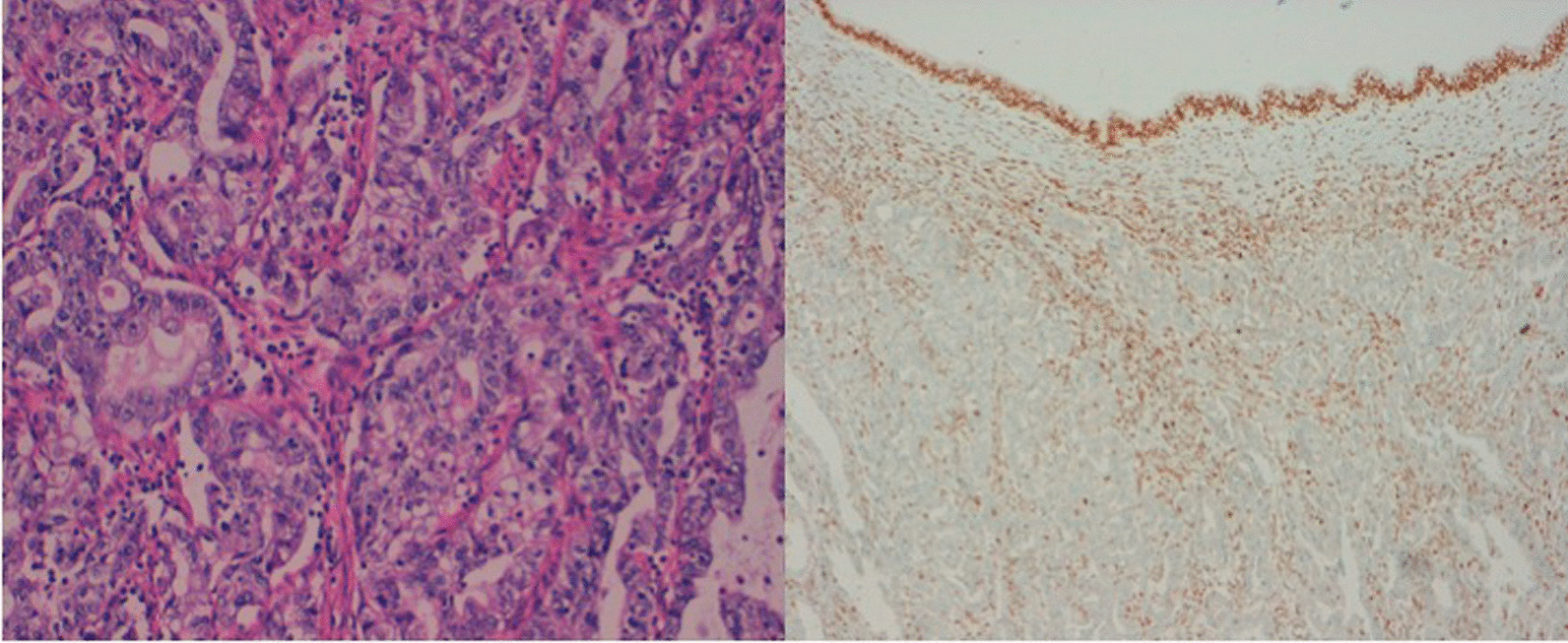
Histological sample of endometrial carcinoma. Histological specimen showing a clear cell endometrial carcinoma on hematoxylin and eosin staining (× 20 magnification) and loss of MSH2 on immunohistochemistry staining (× 10 magnification)

Histology from the uterine biopsy resulted in the diagnosis of a clear cell adenocarcinoma of the endometrium (shown in Fig. 2). P53 was overexpressed with a deficiency in MSH2-repair-protein in immunohistochemistry (shown in Fig. 2) with a preserved expression of MSH6.

A CT scan of thorax and abdomen and a pelvic MRI showed no distant metastasis or locoregional disease. Colonoscopy and gastroscopy showed no pathologic findings.

Four weeks postpartum we performed a laparotomy with hysterectomy, bilateral salpingo-oophorectomy, infragastric omentectomy, pelvic and paraaortal lymphadenectomy. The final pathology revealed a pT2, pN0 (0/45 lymph nodes), L1, V0, Pn0 poorly differentiated clear cell adenocarcinoma of the endometrium progressing to the cervix (UICC-stadium II).

After a prolonged postoperative course, restitution and final wound closure, chemotherapy with carboplatin (AUC5) and paclitaxel (175 mg/m^2^ body surface area) was started two months after the oncological surgery and five cycles were applied every three weeks. A planned sixth dose was not given due to neutropenia, diarrhea and a urinary tract infection. External beam radiotherapy (45 Gy) of the pelvis followed with a simultaneous integrated boost on the former tumor region (50 Gy).

Six months after completion of adjuvant systemic and radiation therapy, the patient presented with signs of an ileus. Multiple adhesions were found in re-laparotomy where an ileum segment was excised and a metastasis measuring 3.6 cm infiltrating the subserous tissue was confirmed. Imaging did not show any further metastatic lesions. After a new prolonged postoperative course with vacuum surgery therapy, the patient is currently undergoing immune checkpoint inhibitor therapy with pembrolizumab.

## Discussion and conclusion

This is a case with diagnosis of clear cell type II endometrial cancer in pregnancy, namely after delivery via caesarean section. This presented EC in pregnancy is unique due to its clear cell histology and its diagnosis with a live birth. This combination is exceptional in the scarce entity of pregnancy-related ECs and specifically since diagnosis was made at delivery and not following a dilation and curettage. Due to the aggressive tumor properties and dMMR status, the patient was treated with surgery, radiotherapy and adjuvant chemotherapy. She nevertheless developed recurrence and is now under treatment with pembrolizumab according to current guidelines [18–21].

An interesting aspect is, that the diagnosis of a MSH2 mutation, leading to a MSI-high/dMMR status, was initially made through fetal whole exome sequencing. Amniocentesis with subsequent determination of fetal karyotype is a standard procedure if ultrasound-guided suspected fetal anomalies occur. Whole exome sequencing can be furthermore done if conventional testing remains unremarkable [22]. This showed a result, unlikely to be related to the sonographically described fetal cardiac abnormality, but potentially indicating a parental hereditary condition. This MSH2 gene mutation in the fetal genetic testing could have guided towards the rare diagnosis of endometrial carcinoma in the pregnancy, especially in the case of a suspected fibroid in the lower uterine segment. As mentioned, alterations in MSH2 gene lead to a deficiency in the mismatch repair system and aggregation of mutations can cause carcinogenesis. Suspicion for EC in pregnancy could lead to the diagnostic dilemma of potential adverse peritoneal tumor spillage [18] through uterotomy necessary for delivery via caesarean section. Tissue was only obtained at delivery and was originally assessed as a necrotizing fibroid, but emphasizes the need for confirmation by biopsy of suspicious masses also in pregnancy. In this situation, presumption of possible uterine malignancies is difficult to verify through biopsy without risking the ongoing pregnancy and potential tumor spread through the access route. This is specific to uterine masses since other gynecologic and non-gynecologic tumors can be safely detected through intervention, biopsy or surgery.

While EC in pregnancy is a rare entity [23], it is mostly found at dilation and curettage for miscarriage in the first trimester or in the postpartum period due to prolonged bleeding. The literature shows only four cases that were diagnosed during pregnancy or at delivery [5, 24–26]: Wall describes a case where a biopsy was taken from a bleeding cervical lesion at 5 months of gestation, leading to the later diagnosis of adenocarcinoma of the uterine corpus [26]. Schammel et al. performed a caesarean section for premature rupture of fetal membranes and intraoperative endometrial curettage resulted in diagnosis of a G1 endometrioid adenocarcinoma [25]. Shiomi et al. performed abdominal hysterectomy for diagnosis of placenta accreta with 35 weeks of gestation and histopathology revealed a G1 endometrioid adenocarcinoma [5]. Most recently, a G2 endometrioid adenocarcinoma was diagnosed after examination of the placenta following premature rupture of membranes by Maeda and colleagues [24]. No other EC types besides endometrioid adenocarcinoma were described in these cases.

Type I endometrial carcinoma is more common in premenopausal women than type II non-endometrioid carcinomas including clear cell carcinoma [16]. We could only identify one pregnancy-related case with clear cell endometrial carcinoma: Ohwada and colleagues described a clear cell adenocarcinoma simultaneous in uterus and ovary. In contrast to our case, this was diagnosed 17 months postpartum [27].

Most pregnancy-related endometrial carcinomas are low grade carcinomas, since only three high grade cases can be found in the literature: Laing-Aiken et al. diagnosed a G3 adenocarcinoma 6 weeks postpartum via dilatation and curettage for suspected retained products of conception [4]. Kodoma et al. reported a G3 adenosquamous carcinoma seven months postpartum [28] and Ota et al. described an extensive progressing G3 tumor in the pregnancy which was diagnosed as atypical polypoid adenomyoma but managed conservatively before conception [29]. More commonly, EC in pregnancy or puerperium are well-differentiated (G1) endometrioid adenocarcinomas with minimal invasive disease [4–7, 30]. These tumors seem to have a good prognosis, similar to tumors without association to pregnancy. Endometrial carcinomas with serous, clear cell or undifferentiated histology are defined as high grade without histologic grading [19, 31]. Thus, the presented clear cell endometrial cancer is a high grade tumor.With dMMR EC in pregnancy being a rare entity, a universal screening for MSI through fetal genetic testing is not reasonable. With growing diagnostic tools in prenatal care and possible increasing number of genetic results, an interdisciplinary approach and thorough genetic counseling is necessary to improve women’s health in pregnancy. If dMMR is detected prenatally, clinical and imaging exams could be considered due to association with Lynch syndrome and breast cancer in pregnancy [32] but most importantly EC since this is the most common tumor with dMMR [33]. Radiomic profiling from MRI [34] or potentially ultrasound exams [35] showed to refine tumor characteristics and this might replace molecular profiling for treatment guidance in the future after confirmation of cancer. However none of the non-invasive techniques have been validated in pregnancy.

If masses are suspected, especially with a mutation in a known high-risk gene, biopsy at or immediately after delivery must be performed. More than 90% of cases of dMMR EC are endometrioid [33, 36]. In clear cell EC only 20% are suspected of being deficient in MMR [33]. So even with the known MSH2 mutation and ultrasound evidence of a uterine mass, we could not have expected a clear cell carcinoma in pregnancy.

Besides genetic alterations, high estrogen levels facilitate endometrial carcinomas. This can result from obesity, infertility, polycystic ovarian syndrome, anovulatory cycles among others [28]. A history of gestational diabetes also doubles the risk for endometrial cancers [37]. The presented patient was diagnosed with gestational diabetes in two earlier pregnancies and had an elevated BMI. Both existent risk factors do favor type I, and especially dMMR subtypes, but not type II EC [38, 39]. The patient exhibited only few risk factors for EC, but they are not even associated with the diagnosed type II endometrial carcinoma.

Endometrial carcinoma is a rare tumor entity in pregnancy. This case shows, EC should be included as a differential diagnosis for fibroid-like tumors in patients with risk factors like bleeding, obesity, gestational diabetes and especially with proven mutations. Suspicion of abnormal masses in the pregnancy should be followed up with obtaining a histology sample latest at delivery to not further delay diagnosis of possible aggressive carcinomas. However, no diagnostic algorithm can be deducted as this case report is limited due to the rare presentation of a gynecological malignancy in pregnancy.

We describe a case of clear cell endometrial adenocarcinoma diagnosed during cesarean delivery in the 27th week of pregnancy through obtaining tissue of a suspicious uterine mass. This should increase awareness to include rare endometrial cancer as a differential diagnosis of uterine masses in pregnant women, especially with risk factors such as bleeding or underlying genetic aberrations.

## Data Availability

Since this is a case report, all data is confidential due to protect the patient`s identity. The medical records are accessible by all authors. Information regarding the literature search can be requested from F.W.

## Abbreviations

APGAR: Apgar Score
AUC5: Target area under the concentration versus time curve
BMI: Body mass index
cm: Centimeters
CT: Computer tomography
dMMR: Mismatch repair deficient
EC: Endometrial carcinoma
ECs: Endometrial carcinomas
g: Grams
G1: Well differentiated, low grade
G2: Moderately differentiated, intermediate grade
G3: Poorly differentiated, high grade
Gy: Gray
i.m.: Intramuscular
i.v.: Intravenous
m^2^: Square meters
mg: Milligrams
mm: Millimeters
MMR: DNA mismatch repair system
MRI: Magnetic resonance imaging
MSI: Microsatellite instability
p53: Tumor protein 53
PAP: Papanicolaou
POLE: Polymerase-epsilon-mutated
UICC: Union for International Cancer Control
